# Case Report: Papillary Lesions at the Mouth Floor May Mimic Sialadenoma Papilliferum

**DOI:** 10.3389/pore.2022.1610352

**Published:** 2022-07-14

**Authors:** Dawool Han, Eunae Sandra Cho, Jiho Park, Dongwook Kim

**Affiliations:** ^1^ Department of Oral Pathology, Oral Cancer Research Institute, Yonsei University College of Dentistry, Seoul, South Korea; ^2^ Department of Oral and Maxillofacial Surgery, Yonsei University College of Dentistry, Seoul, South Korea

**Keywords:** salivary gland, tumor, sialadenoma papilliferum, oral pathology, papillary lesion, oral cavity, oromaxillofacial

## Abstract

Salivary gland tumor Sialadenoma papilliferum (SialP) clinically resembles papillary epithelial lesions, such as squamous papilloma (SqP) or verrucous leukoplakia. Pathological sampling including an adequate depth of both the mucosa and submucosa layer is required for discrimination between the diseases. Though ductal proliferation in the submucosa is characteristic in SialP, papillary lesions arising at the mouth floor, specifically near the ductal orifice, are more problematic. Salivary gland ductal ectasia, along with the overlying papillary hyperplasia, may mimic the biphasic tumorous growth pattern of SialP, making discrimination extremely difficult. Further cellular dysplasia in the papillary mucosal lesion raises the possibility of malignant transformation in a known benign lesion, SialP. Herein, we present a case of SqP at the mouth floor which mimicked both clinical and pathological features of SialP and compared it with a definite case of SialP. Moreover, we discuss major differential points that clinicians and pathologists should consider during diagnosis of oral papillary lesions arising near the salivary glands.

## Introduction

The majority of oral solitary papillary lesions are derived from the surface squamous epithelium. Depending on the size, shape and amount of keratinization, the papillary lesions may be clinically assumed as squamous papilloma, verruca vulgaris, verruciform xanthoma, verrucous carcinoma or papillary squamous cell carcinoma. Among these, squamous papilloma (SqP) is a benign epithelial tumor prevalent in the oral cavity. The characteristic clinical features of a solitary exophytic lesion with pink to whitish multiple papillary projections, in many cases pedunculated (meaning an outgrowing lesion with a “stalk” connected to the surface), and limited growth within a diameter of 1 cm easily leads to the impression of SqP.

However, in papillary lesions that occur over or near the minor salivary glands or major salivary gland ductal structures, a completely distinct entity called sialadenoma papilliferum (SialP) must be considered for differential diagnosis. SialP is a rare benign salivary gland tumor first reported in the literature in 1969 (1). It commonly occurs in the soft and hard palate junction and buccal mucosa but can occur wherever the salivary glands are located, including the upper airway (2-6). It is clinically indistinguishable from SqP because it presents as a single papillary or verrucous exophytic mass, usually less than 1 cm in diameter, but in a few cases larger than 2 cm (6-8). Fortunately, SialP has a striking biphasic histopathologic growth pattern. The surface keratinized or non-keratinized squamous epithelium of SialP proliferates in a papillary manner with fibrovascular cores similar to SqP, whereas the downward growth of tumorous salivary ductal epithelium is unique to SialP. This ductal component of dilated or multi-cystic cuboidal to columnar structures with micropapillary luminal projections is relatively definite, but reactive dilation of ductal structures beneath SqP arising in the salivary gland orifice can show a similar appearance. Herein, we report a case of SqP which arose on the submandibular gland orifice with accompanying reactive ductal dilation, making it difficult to pathologically discriminate from SialP. We will discuss critical differences between the diseases by comparing two separate cases, oral SqP and SialP, respectively.

## Case Report

**FIGURE 1:**
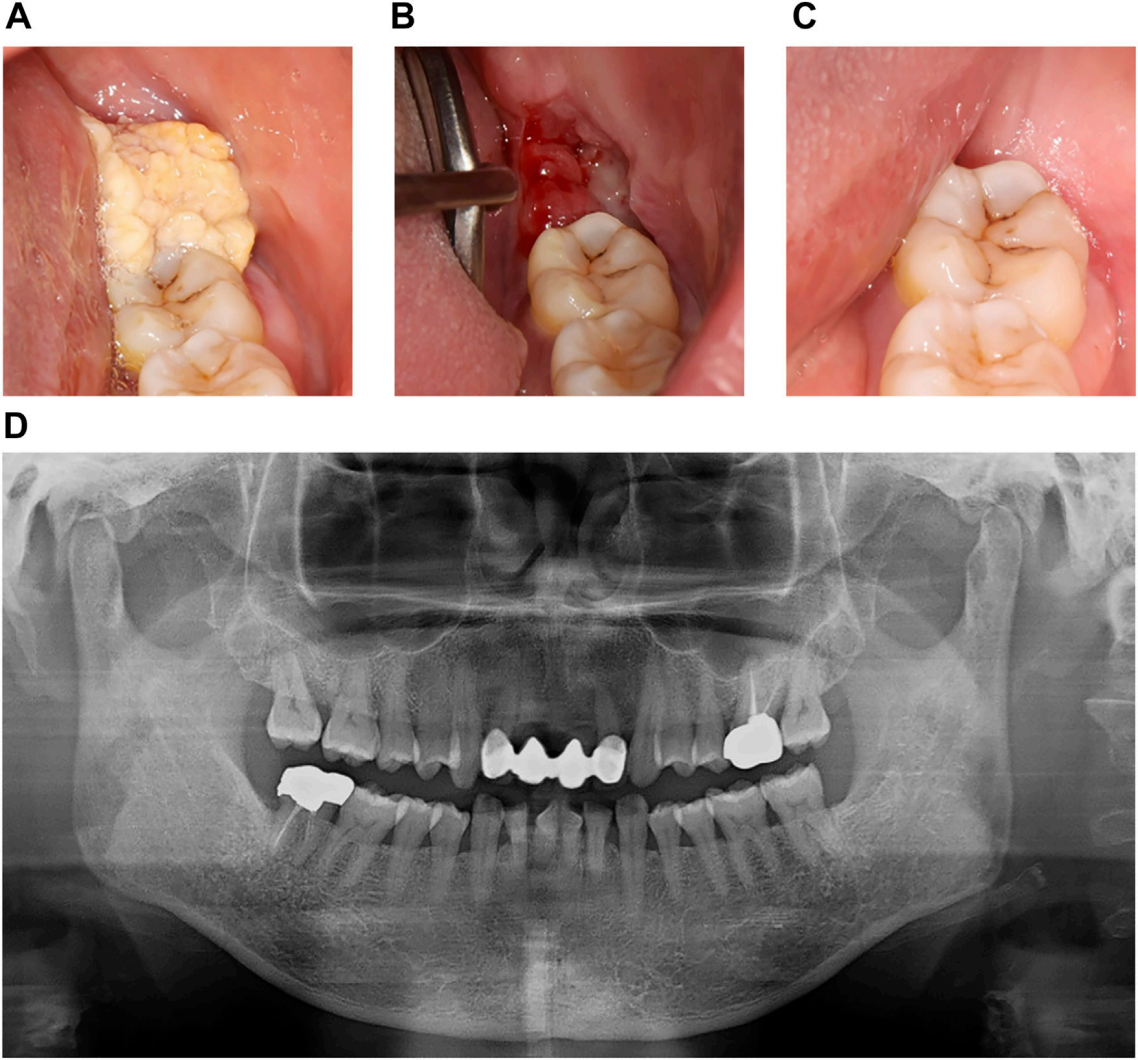
Clinical images and panoramic radiograph of the left mandibular lesion of case #1. **(A)** First visit. Verrucous exophytic, whitish-to-yellowish mass was observed on the left retromolar trigone area. **(B)** 4 weeks after the first visit and excisional biopsy. Papillomatous lesion, about 3 mm in diameter, recurred on the excision site. **(C)** 22 weeks after the first visit. There was no remarkable recurred lesion on the left retromolar trigone area. **(D)** Panoramic radiograph at first visit. Cortical erosion or bony destruction of mandible was not observed below the papillary lesion.

**FIGURE 2:**
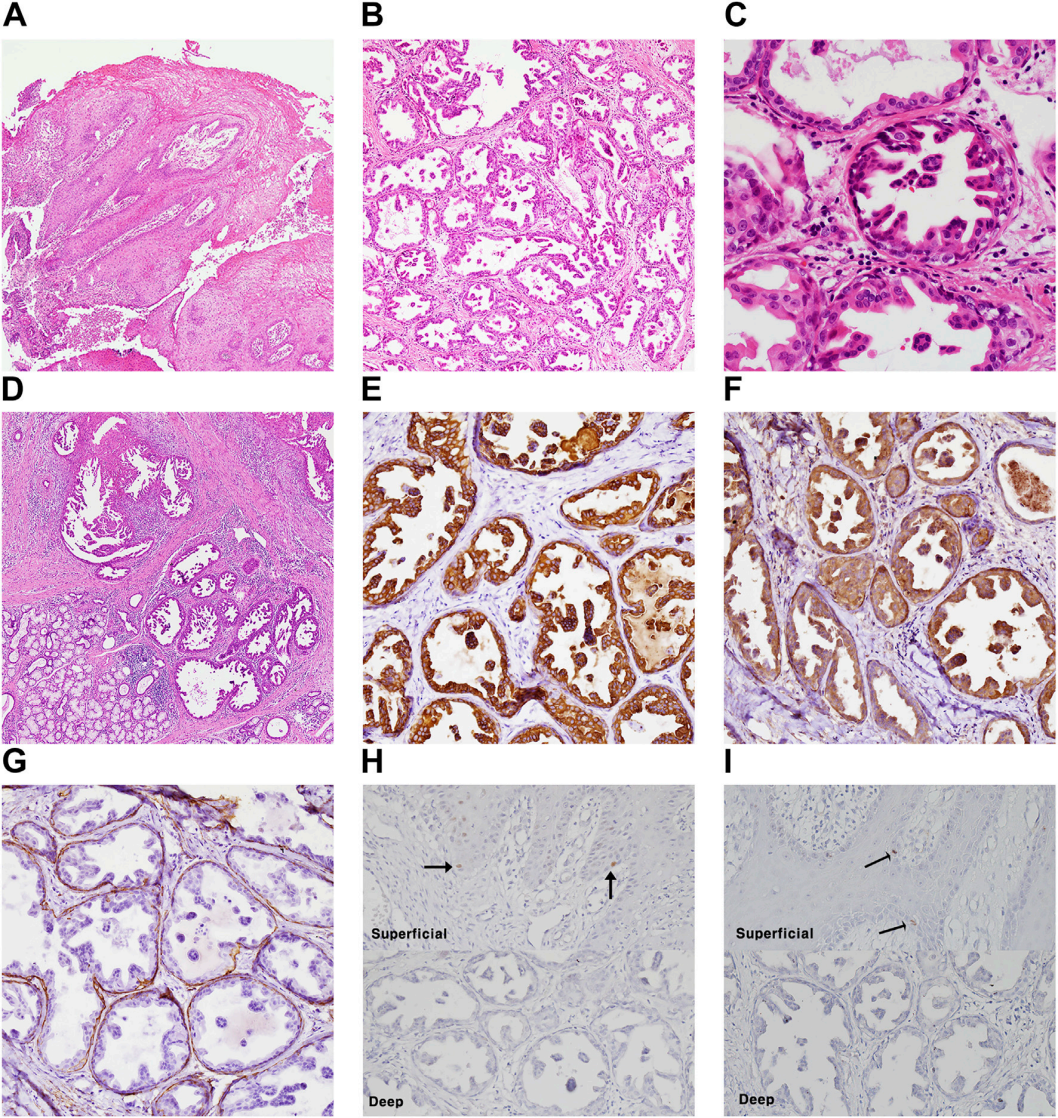
Histopathologic examination and molecular pathologic results of surgical specimen of case #1 (classic SialP). **(A)** Papillary proliferation of surface mucosal epithelium, which is supported by fibrovascular core (Hematoxylin and eosin, ×40). **(B)** Deep portion of tumor is composed of proliferating salivary ductal epithelium (Hematoxylin and eosin, ×40). **(C)** The proliferating ductal epithelium is cuboidal shape with eosinophilic cytoplasm and centric nucleus. It shows papillary folds into the luminal space. Also, there is one layer of flattened cell on the basal side, known as basal cell (Hematoxylin and eosin, ×400). **(D)** The recurrent lesion after first excision showed the same features of ductal proliferation as the previous excised SialP (Hematoxylin and eosin, x40). **(E–G)** Proliferating ductal epithelium showing positive on CK7(E) and S-100(F). Basal cells are positive on smooth muscle actin **(G)** (immunohistochemistry staining, ×200). **(H,I)** A few scattered basal cells showed nuclear positive on p53 **(H)** and Ki-67 **(I)** in the superficial papillary mucosal epithelium (black arrow), not in the deep adenoma portion (immunohistochemistry staining, ×200).

Case #1 (Classic SialP at the retromolar trigone) A 53-year-old Asian female visited the Department of Oral and Maxillofacial Surgery at Yonsei Dental Hospital, complaining of a whitish painless growing mass on the left retromolar trigone (RMT) which had started a month before. According to the patient, there had been no pain. She was on medication for rheumatoid arthritis that had been prescribed by a local clinic. There were no other specific past medical or dental histories associated with the lesion. The lesion was a whitish-to-yellowish verrucous mass on the left RMT area (Figure 1A). There were no erythema, ulceration or adjacent lesions. There were no tenderness or bleeding tendency on palpation. The size of the lesion was approximately 3.0 × 2.0 cm. There were no abnormalities on extraoral examination. Bony destruction was not detected beneath the mass on a panoramic exam (Figure 1D). Clinically, the impression was either SqP or verrucous carcinoma. Excisional biopsy was done on the first visit. The specimen was submitted as two components: a superficial exophytic mass and the underlying tumor base. Histopathologic examination revealed that the lesion was composed of two parts with significantly distinct pathologic characteristics. The superficial exophytic mass was composed of papillary proliferations of keratinized stratified squamous epithelium originated from the mucosa, consistent with a SqP (Figure 2A). The deep portion of the lesion showed proliferations of cuboidal ductal epithelium with eosinophilic cytoplasm, but without nuclear atypia (Figures 2B,C). The tumorous ductal epithelium extended into and merged with the superficial papillary mucosal epithelium in a continuous manner. Immunohistochemical (IHC) staining revealed that the tumorous ductal epithelium in the deep component was composed of cytokeratin 7 (CK7) (+), S-100 (+), smooth muscle actin (SMA) (−) cuboidal cells with surrounding SMA (+) basal cells (Figures 2E–G). The superficial tumorous component did not display diffuse CK7 staining, distinct from the deep component. Moreover, p53 and Ki-67 protein expression were positive in only a few scattered basal cells of the superficial papillary epithelium on IHC staining (Figures 2H,I). On the basis of the microscopic characteristics, a diagnosis of sialadenoma papilliferum was established. Since residual tumor was suspected in the basal area, a further excision was done. Four weeks after excision of the lesion, the patient complained about a newly growing mass on the removal site (Figure 1B). Computed Tomography (CT) and Magnetic Resonance Imaging (MRI) were taken for further evaluation of the operation site. There was a mild bulging and enhancement at the left retromolar trigone region with underlying bony erosion, suggesting post-operative changes or presence of a residual tumor. There was a 3 mm-sized papillary mass on the formerly excised site. The lesion was excised along with normal adjacent tissue. Histopathological examination revealed residual adenoma structures as seen in the first excision (Figure 2D). Sixteen weeks after the second excision, there was no recurrence on the RMT area (Figure 1C). Nine months after the second excision, there were no signs of recurrence.

**FIGURE 3:**
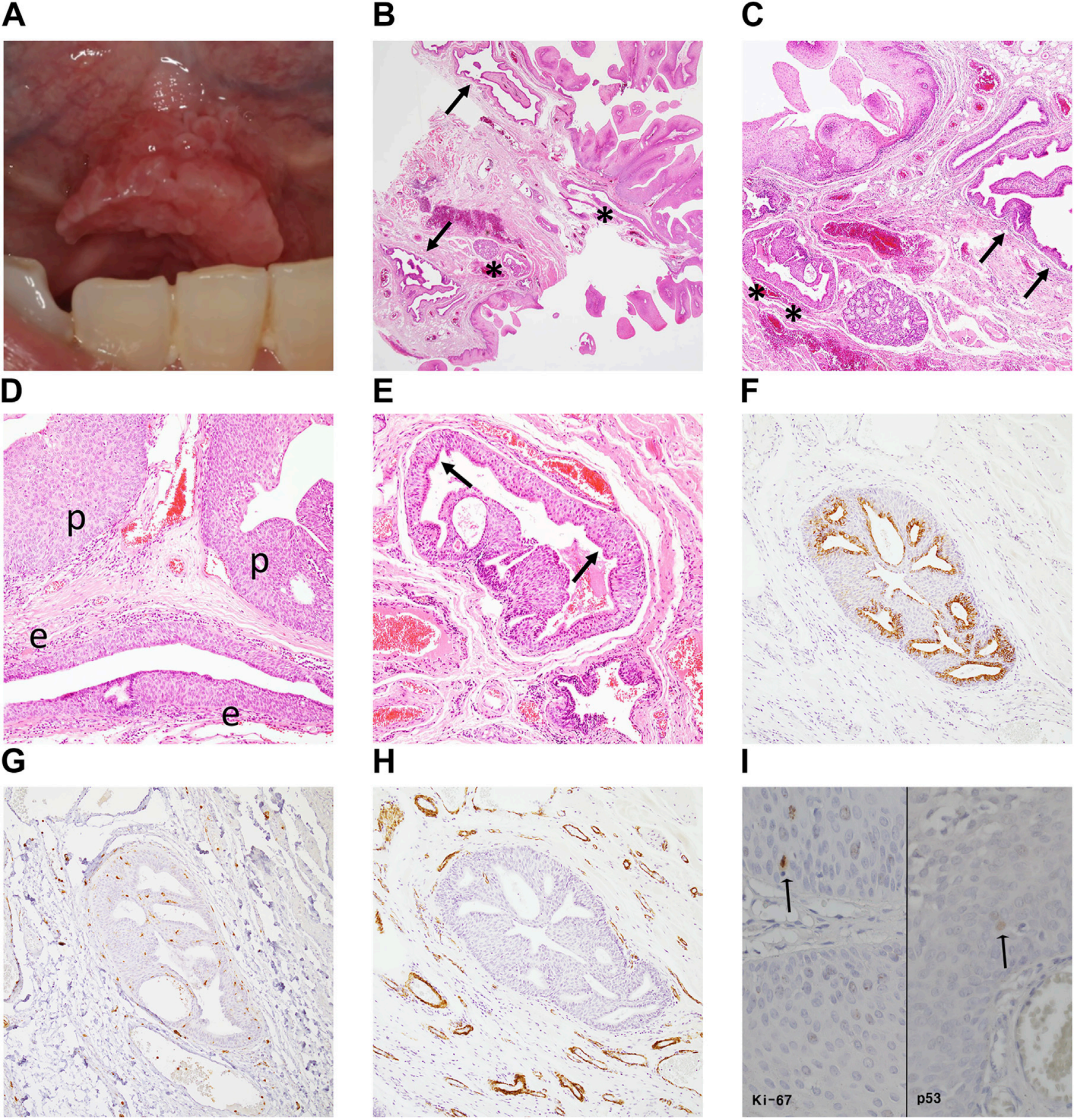
Clinical image, histopathologic examination and molecular pathologic results of the surgical specimen of case #2 (SqP mimicking SialP). **(A)** There was a pinkish exophytic papillary mass on the floor of mouth including lingual frenum. It looked like squamous papilloma, clinically. **(B,C)** Papillary proliferation of the surface mucosal epithelium supported by fibrovascular core with underlying “superior” hyperplastic ductal structures (asterisks) and “inferior” dilated and tortuous excretory ducts (arrows) (Hematoxylin and eosin, ×12.5, ×40). **(D)** Mucosa region: Superficial papillary mucosal epithelium (p) showed mild atypia. The underneath excretory duct is hyperplastic (e) (Hematoxylin and eosin, ×100). **(E)** Superior submucosa regions: hyperplastic epithelium of the excretory duct with microcystic-like changes. There is a layer of ciliated columnar epithelium (luminar layer, arrows) on the hyperplastic squamous stratification (Hematoxylin and eosin, ×100). **(F)** Only the luminal columnar epithelium layer was positive for CK7 staining (Cytokeratin 7, ×100). **(G)** S-100 was diffusely negative in the superior hyperplastic excretory ductal epithelium (S-100, ×100). **(H)** Unlike in SialP, the ductal epithelium was not surrounded by SMA (+) myoepithelial cells, a histologic finding consistent with normal excretory ducts (smooth muscle actin, ×100). **(I)** Minimal p53 and Ki-67 expression were observed in the epithelial basal layer. That is, the atypical proliferation was judged to be a reactive change rather than a precancerous change (immunohistochemistry staining, ×400).

Case #2 (SqP mimicking SialP at the mouth floor) A 47-year-old Asian male visited the Department of Oral and Maxillofacial Surgery at the Yonsei Dental Hospital complaining of a papillary mass under the tongue that had occurred a month before without a specific event. There was no pain or other remarkable symptoms. He had a past medical history of treated tuberculosis. On clinical examination, an exophytic verrucous mass was observed from the floor of mouth to the lingual frenum (Figure 3A). It had a maximum diameter of about 1.5 cm, and showed a pink surface color similar to the surrounding normal mucosa. In addition, the lesion was soft on palpation, with no observable tenderness or induration. It was excised and submitted for histopathologic examination under a clinical impression of SqP. Low-power histopathological examination revealed an exophytic papillary mucosal epithelium with an underlying adenomatous submucosa compartment (Figure 3B). The submucosa compartment was composed of a “superior” hyperplastic and microcystic-like portion and an “inferior” dilated and tortuous ductal portion (Figure 3C). The superficial papillary mucosa had focal atypical features such as bulbous rete ridges and disorganized basaloid proliferation (Figure 3D). In the superior submucosa portion, the overall epithelial structure was adenomatoid, with hyperplastic squamous epithelium overlined by a layer of ciliated columnar epithelium (Figure 3E). Microcystic or microductal structures were seen within the superior hyperplastic ductal epithelium. In the inferior submucosa portion, indistinct micropapillary-like projections were seen within the dilated columnar ductal epithelium (Figure 3C). The inferior portion lacked any evidence of tumorous adenomatous proliferation. Differential diagnosis between SqP and SialP was required. IHC staining revealed that CK7 was only positive in the ductal epithelium of the inferior portion and in a single layer of luminal epithelium in the superior submucosa portion (Figure 3F). Importantly, diffuse S-100 expression and abluminal SMA staining typically seen in SialP were not seen in this lesion. The superior submucosa portion did not show SMA (+) myoepithelial cells, consistent with a general excretory duct histology rather than the salivary gland tumor, SialP (Figure 3H). These findings indicated a definite tumorous papillary growth in the superficial mucosa with extension to the superior excretory duct and additional presence of reactive ductal dilation.

Although the overall lesion did not present definite cellular pleomorphism or loss of polarity, since the epithelial basal cells showed bulbous architecture, we evaluated the lesion for the possibility of early-stage malignant transformation. The IHC results showed that the Ki-67 proliferation index and p53 expression were minimal and localized at the basal layer in both the lesional epithelium and adjacent normal mucosa (Figure 3I). This pattern suggested that the mild epithelial atypia in Case #2 was more likely to be benign and reactive rather than premalignant, as occasionally seen in SqPs. The final diagnosis was SqP with mild atypia and ductal extension.

## Discussion

Despite the similar clinical features of SialP and papillary lesions of the squamous mucosa such as SqP, histological discrimination is generally not a problem because of the submucosal glandular proliferation in addition to the common squamous papillary growth (7, 9-11). The problem is that SqP arising in the salivary gland duct orifice, the opening, can pathologically mimic SialP, confusing the pathologist and leading to misdiagnosis. The possibility of misdiagnosis in papillary lesions in this case has been mentioned in the literature. Authors have argued that some presented case reports of SialP should be considered papillary epithelial lesions (9, 12). Such cases, located at the mouth floor, had a superficial papillary lesion with a controversial submucosal ductal component (9, 12). We agree that it may be more appropriate to consider the submucosal components as tortuous ductal dilation and ectasia rather than true tumorous ductal proliferation based on given histopathological figures.

This argument can be critical because both mouth floor SialP cases had atypical cellular features, raising the question whether SialP may present malignant transformation. Since SialP is a very rare tumor, only 89 cases have been reported in the literature, and evidence of atypical clinical prognosis is scant (7). Although the tumor is generally considered benign, issues of potential malignant transformation have been suggested (12-16). We do not have sufficient data to analyze the malignant potential of SialP but do believe that some cases of reported SialP with cellular atypia may actually be dysplastic papillary epithelial lesions with underlying ductal ectasia and ductal *in situ*. Papillary epithelial lesions may have a various spectrum of cellular atypia which may extend into the superficial salivary duct when arising at the orifice. Mild basal cell dysplasia is a common feature in benign SqP, not a sign of malignant transformation (17). Other truly precancerous or cancerous changes can be seen in verrucous leukoplakia, carcinoma *in situ* or papillary squamous cell carcinoma. Other authors have suggested that papillary precancerous or cancerous lesions with ductal extension have been frequently misdiagnosed as SialP (9, 12), which may disrupt the statistics of malignant potential and clinical prognosis in SialP. To further address this issue, we applied immunohistochemical analysis to two of our cases in order to effectively discriminate between SialP and papillary epithelial lesions.

We propose several histopathologic points that may be effective in distinguishing SialP from papillary epithelial lesions, representatively, SqP. Case #2 was a SqP with mild basal dysplasia which slightly extended into the underlying ductal ectasia component. These histological features caused confusion with SialP at first sight, which was later resolved by immunohistochemistry. Markers of the salivary gland ductal epithelium and myoepithelial cells were combined to assess the micro-histological features of the submucosal component. The stain panel of cytokeratin 7 (CK7), S-100, and smooth muscle actin (SMA) made it possible to distinguish whether the ductal proliferation was true neoplastic growth as in SialP or dilation of the normal duct. In the case of SialP, ductal proliferation shows diffuse and strong positivity of CK7, while it is expressed only in the luminal columnar cells of the duct in the latter case (7, 18-20). In addition, S-100 and SMA are positive in basal cells in SialP but absent in the striated and excretory normal and dilated duct (21). Other than the immunohistologic differences, the dilated duct of Case #2 lacked the typical double cuboidal/columnar cell layer with micropapillary components, instead showing a thicker tortuous layer of squamous-like epithelium (7, 9, 19).

Importantly, the limited protein expression of p53 and Ki-67 in Case #1 and #2 suggested both lesions were benign tumors without direct evidence of malignant potential. Squamous cell carcinoma, the most prevalent type of oral cancer, and high grade precancerous lesions derived from oral mucosa frequently have an aberrant *TP53* mutation profile which may present as a gain-of-function positive IHC pattern during and after malignant transformation (22-24). The role of *TP53* gene mutations in salivary gland tumors is less conspicuous than in oral squamous cell carcinomas (25-27). Only one previous case report implied the malignant potential of oral SialP by increased p53 expression in the high grade area of the tumor, but our SialP diagnosed case (Case #1) did not show significant patterns of increased p53 expression (14). In most previous studies, malignant transformation-related assessment in SialP has been limited to histo-morphological evaluation. As in the case of other salivary gland tumors, long-term follow-up data and further research into molecular pathology markers are needed to define the biological potential of SialP.

Recent studies have determined that SialP is a true neoplastic region with mutual genetic alterations (7, 28, 29). Hisieh and Nakaguro recently analyzed the genetic alterations of the papillary cystic tumor of salivary gland duct including SialP and confirmed that a significant portion of BRAF V600E mutation were in SialP (28, 29). However, different results were obtained from the SialP-like lesions not accompanied by exophytic papillary proliferation of the mucosa, which was expressed as exophytic ductal papilloma or SialP-like intraductal papillary tumor (SP-IPT) in each report (28, 29). Also, HRAS Q61R mutation was observed in one of the eight SialP cases (12.5%) and in one of the two SP-IPT cases (50%) (29). Additional reports have confirmed BRAF mutation in a majority of SialP, as analyzed by Sanger sequencing or IHC staining (3, 7). These BRAF and HRAS mutations have already been identified in syringocystadenoma papilliferum. These two tumors were considered counterparts based on their morphological similarity, genetic profiling now also highlights the similarity of the two entities (30, 31). In cases where morphological and IHC staining features are ambiguous in diagnosing SialP, mutation profiling may be used as an auxiliary test to detect these genetic mutations.

However, SialP is known as a benign lesion with excellent prognosis. In reports with sufficient follow-up data, only 2 cases of recurrence have been recorded, with 4 cases being reported to have malignant transformation (Table 1) (7, 12-16, 18, 32, 33). Two of these four have been questioned by several authors (9, 12). Oral papillary lesions with dysplastic characters such as papillary squamous cell carcinoma and dysplastic verrucous hyperplasia, can extend into the salivary gland duct and mimic SialP. Therefore, papillary lesions arising at the salivary gland opening, particularly the mouth floor, require caution during both clinical and histologic diagnosis. Specific immunohistochemistry applications can aid proper discrimination between the two separate entities. These considerations are essential during assessment of malignant transformation and clinical prognosis of SialP.

**TABLE 1:** Two reported cases with recurrence of benign SialP and five reported cases of malignant transformation in SialP (1978–2021).

Recurrent cases					
Reference	Age	Sex	Location	Clinical features	Recurrence
(33)	78	F	Junction of soft and hard palate	Firm warty papule	Recurred after 3 years
(32)	67	F	Buccal mucosa	Non-pedunculated exophytic mass	Recurred after 3 years

Malignant transformed cases						
Reference	Age	Sex	Location	Clinical features	Associated malignancy	Follow-up
(13)	62	M	Soft palate	Exophytic papillary mass	Squamous cell carcinoma	Mutiple recurrence
(14)	79	F	Junction of soft and hard palate	Exophytic pink-white papillary mass	Epithelial-myoepithelial carcinoma	Exp—1 month (hemorrhage)
(15)	30	M	Floor of mouth	Exophytic, slightly papillary lesion	Carcinoma *in situ* in the mucosal component of SialP	NED—8 months
(16)	82	F	Base of tongue	Exophytic papillary mass	Mucoepidermoid carcinoma	N/A
(12)	67	F	Retromolar trigone	Warty-surfaced bulky mass	Dysplastic features in the glandular component of SialP	NED—3 years

Exp, Expired; NED, no evidence of disease; N/A, not available.

## Data Availability

The original contributions presented in the study are included in the article/supplementary material, further inquiries can be directed to the corresponding author.
